# Evaluation of the maxillary sinus in panoramic radiography—a comparative study

**DOI:** 10.1186/s40729-015-0015-1

**Published:** 2015-07-10

**Authors:** Johann Malina-Altzinger, Georg Damerau, Klaus W Grätz, PD Bernd Stadlinger

**Affiliations:** 1 Clinic of Cranio-Maxillofacial Surgery, University Hospital of Bern, Freiburgstrasse 4, 3010 Bern, Switzerland; 2 Clinic of Oral Surgery, Center of Dental Medicine, University of Zurich, Plattenstrasse 11, 8032 Zurich, Switzerland; 3 Former Head of the Department of Cranio-Maxillofacial and Oral Surgery, University of Zurich, Plattenstrasse 11, 8032 Zurich, Switzerland

**Keywords:** Panoramic radiography, Cone beam computed tomography, Maxillary sinus, Inter-imaging method differences, Inter-examiner reliability, Intra-examiner reliability

## Abstract

**Background:**

The aim of this study was to evaluate the validity and the inter- and intra-examiner reliability of panoramic-radiograph-driven findings of different maxillary sinus anatomic variations and pathologies, which had initially been prediagnosed by cone beam computed tomography (CBCT).

**Methods:**

After pairs of two-dimensional (2D) panoramic and three-dimensional (3D) CBCT images of patients having received treatment at the outpatient department had been screened, the predefinition of 54 selected maxillary sinus conditions was initially performed on CBCT images by two blinded consultants individually using a questionnaire that defined ten different clinically relevant findings. Using the identic questionnaire, these consultants performed the evaluation of the panoramic radiographs at a later time point. The results were analyzed for inter-imaging differences in the evaluation of the maxillary sinus between 2D and 3D imaging methods. Additionally, two resident groups (first year and last year of training) performed two diagnostic runs of the panoramic radiographs and results were analyzed for inter- and intra-observer reliability.

**Results:**

There is a moderate risk for false diagnosis of findings of the maxillary sinus if only panoramic radiography is used. Based on the ten predefined conditions, solely maxillary bone cysts penetrating into the sinus were frequently detected differently comparing 2D to 3D diagnostics. Additionally, on panoramic radiographs, the inter-observer comparison demonstrated that basal septa were significantly often rated differently and the intra-observer comparison showed a significant lack in reliability in detecting maxillary bone cysts penetrating into the sinus.

**Conclusions:**

Panoramic radiography provides the most information on the maxillary sinus, and it may be an adequate imaging method. However, particular findings of the maxillary sinus in panoramic imaging may be based on a rather examiner-dependent assessment. Therefore, a persistent and precise evaluation of specific conditions of the maxillary sinus may only be possible using CBCT because it provides additional information compared to panoramic radiography. This might be relevant for consecutive surgical procedures; consequently, we recommend CBCT if a precise preoperative evaluation is mandatory. However, higher radiation dose and costs of 3D imaging need to be considered.

## Background

The development of two-dimensional (2D) panoramic imaging techniques began in the first half of the 20th century, but the first device applying this technology was only described in 1959 [1]. Since then, this radiographic technique has steadily been improved and has become a standard diagnostic tool in a clinician’s daily practice. In parallel, cone beam computed tomography (CBCT), first described in 1982 [2], was introduced as a tool for dental and maxillofacial diagnostics.

The advantages of three-dimensional (3D) CBCT over 2D conventional panoramic tomography include an excellent imaging quality of high-contrast structures like the maxillofacial bone anatomy, no geometric distortion, and no superimposition of surrounding anatomic structures [3]. The advantages of panoramic radiography, on the other hand, are comparatively low-radiation doses, its general availability, and the comparatively low costs. Further, it is especially useful in the initial diagnostic phase of implant planning because it relates information on both dental arches, the inferior alveolar canals, and the maxillary sinuses to its pathologic conditions [4]. However, limitations include the lack of visualization of structures like the bucco-lingual ridge pattern and the visual loss of cortical plates or undulating concavities [5]; moreover, the fact that more than 80 % of measurements from the crest of the residual alveolar ridge to the inferior alveolar canal have errors of more than 1 mm renders panoramic radiography unsuitable as a single imaging source for dental-implant site assessment [4]. Furthermore, it is well known that an average magnification factor of 1.25 can be expected in panoramic radiographs. This demands calibration of the image with the help of a defined reference device when determining the appropriate implant size [6].

Precise assessment of the maxillary sinus is mandatory when planning a lateral or internal sinus floor elevation [7, 8]. It has been claimed that, besides clinical examination, evaluation of the maxillary sinuses is possible by panoramic radiography [9] and CBCT [7, 10]. Though it is known that millions of sinus lift operations were performed with panoramic radiographs without any problems, especially due to the superimposition of different structures, precise assessment of a maxillary sinus finding is difficult in 2D panoramic radiography [11]. This difficulty implies that, as a clinical consequence, patients are often referred to specialists on the basis of a suspected maxillary pathology visualized on a panoramic image. This further requires a CBCT, and the question arises whether a primary CBCT should be performed in cases of maxillary sinus diagnostics instead of an initial panoramic radiography. Moreover, the inter- and intra-examiner variation in the interpretation of 2D radiographs may exceed the variation in imaging techniques and diagnostic yield [12], leading to a rather examiner-dependent assessment of panoramic images.

Therefore, the present study had three aims: the evaluation of the validity (1), the inter- (2), and the intra-examiner (3) reliability of panoramic-radiograph-driven findings of different maxillary sinus conditions which had initially been prediagnosed in CBCT images.

## Methods

The study protocol was approved by the Eidgenössische Expertenkommission für das Berufsgeheimnis in der medizinischen Forschung (Federal expert committee for professional confidentiality in medical research, BAG 035.0001-125/196). All patients have consented to provide their data for research and publication. Prior to this study, a statistical study design was performed, and in a retrospective approach, radiographic images of patients having received treatment at the outpatient department of the Clinic of Cranio-Maxillofacial and Oral Surgery at the University of Zurich were screened. Inclusion criteria were patients having received 2D panoramic radiographs (Cranex, Soredex, Tuusula, Finland) and 3D CBCT (KaVo 3D eXam, Biberach, Germany) showing the maxillary sinus without any surgical procedure in between the two examinations (mean 8.6 days, SD 21 days). Subsequently, possible clinically relevant anatomic variations and pathologies of the maxillary sinus were predefined (nine findings, one unimpaired condition) (Table 1) from a surgical point of view. Twenty-eight patients were selected (63 % males, 37 % females, mean age 47.8 years, range: 20–85 years) according to the conditions shown in Table 1, corresponding to a total amount of 56 maxillary sinuses (one per side).

**Table 1:** Predefined findings of the maxillary sinus

1. Complete opacity	
2. Basal opacity	
3. Foreign body	
4. Oro-antral communication	
5. Basal septum	
6. Polypoid mucosal thickening	
7. Maxillary bone cyst penetrating into the sinus	
8. Fluid level	
9. Status post sinus lift	
10. No finding	

All radiographic images were anonymized and analyzed using the OsiriX Imaging software (version 5.0.2) and a monitor with a display resolution of 1680 × 1050 pixel. Image manipulation through change of brightness, zoom in and out, and rotation was used when needed. No time limit was appropriated, and all observations were performed in the same room under comparable light conditions. Findings of the maxillary sinus were purely based on radiographic appearance without using any other additional clinical or histological information. A questionnaire served for recording the diagnoses (Table 1).

The predefinition of maxillary sinus conditions was initially performed on 3D CBCT images by two consultants in a separate evaluation using the questionnaire. The following reconstruction parameters in all three dimensions (sagittal, coronal, axial) were used: voxel edge length 0.4 × 0.4 × 0.4 mm, slice thickness 1 mm. The two assessors were blinded—disagreement was solved by discussion.

### Evaluation of inter-imaging technique differences

The CBCT assessment served as a reference group for later 2D panoramic image diagnostics which were performed separately by the same two consultants more than 6 months after predefinition on CBCT images. An identical questionnaire was used for both 3D and later 2D diagnostics. Inter-imaging technique differences in evaluating the maxillary sinus with 2D or 3D imaging method were analyzed with regard to false-positive and false-negative decisions.

### Evaluation of the inter-observer reliability

Using the same questionnaire, four blinded residents separately performed the evaluation of the 2D panoramic radiographs—two in the first year and two in the last year of training. Agreement between these two rating groups in detecting findings in the maxillary sinus was analyzed for calculating inter-observer reliability.

### Evaluation of the intra-observer reliability

After more than 4 weeks, the same residents newly evaluated all maxillary sinuses on 2D panoramic images. Reassessment served for the calculation of the intra-observer reliability, which means individual agreement between the two elevation runs.

The results were further evaluated with regard to the prevalence of radiographic findings in the maxillary sinus.

### Data analyses and statistical methods

Data was recorded using Excel 2013 (Microsoft) and analyzed using IBM SPSS Statistics for Macintosh, Verion 22.0 (Armonk, NY: IBM Corp.). Collected data was analyzed to demonstrate degree of agreement using the following statistical tests: logistic regression was used to estimate odds ratios. *p* values were calculated using a chi-squared test. Kappa coefficient and McNemar’s test were used for evaluating the reliability between the two consultants analyzing the CBCT scans. Calculated *p* values were considered significant for values <0.05. Descriptive statistics computed means and standard deviation for quantitative variables as well as absolute and relative frequencies for qualitative variables.

## Results

This study analyzed comparative and descriptive data for the evaluation of the maxillary sinus. Significant differences in the detection of ten predefined findings between 2D and 3D imaging methods were calculated. Furthermore, the degree of agreement in detecting the ten conditions on the panoramic radiographs was measured between different observers and within the same observer. The radiographic images of 28 patients (56 maxillary sinuses, 1 per side) were examined. One patient had to be excluded from the study as the radiographic finding on the CBCT did not clearly match any of the predefined ten conditions.

### Differences between CBCT- and panoramic-radiograph-driven evaluations of the maxillary sinus

**Fig. 1:**
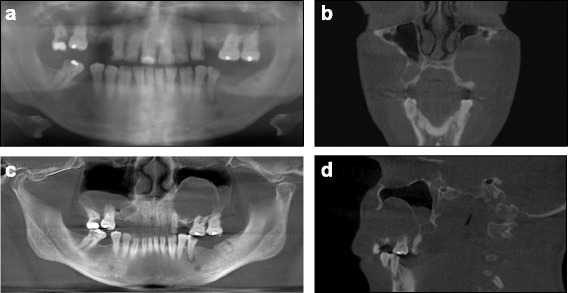
Radiographic findings of patient 26: basal opacity, maxillary bone cyst penetrating into the sinus; **a** panoramic radiography (Soredex, Cranex); **b–d** CBCT images (KaVo 3D eXam): coronal plane (**b**); panoramic reconstruction view (**c**); CBCT sagittal plane, left sinus (**d**)

As illustrated by Table 2, the results of the present study demonstrate that in panoramic-radiograph-driven diagnosis a “no finding” was selected in a quite similar way as if CBCT was used (*p* = 0.803, odds ratio (OR) = 1.220). The difference between the two imaging methods was significant solely for maxillary bone cyst penetrating into the sinus (*p* = 0.032). The estimated OR of this specific finding was significantly lower than 1 (OR = 0.275). No significant differences between 2D and 3D imaging methods were found for the detection of a complete opacity (*p* = 0.998), a basal opacity (*p* = 0.714), a foreign body (*p* = 0.571), an oro-antral communication (*p* = 0.998), a basal septum (*p* = 0.911), a polypoid mucosal thickening (0.123), a fluid level (*p* = 0.253), and a status post sinus lift (*p* = 0.998) (Table 2; Fig. 1).

**Table 2:** Comparison of panoramic radiography and CBCT in detecting ten different conditions of maxillary sinus

	*p*	OR
Complete shadow	0.998	^a^
Basal shadow	0.714	0.822
Foreign body	0.571	1.831
Oro-antral communication	0.998	^a^
Basal septum	0.911	0.945
Polypoid mucosal thickening	0.123	0.376
Maxillary bone cyst penetrating into the sinus	0.032*	0.275
Fluid level	0.253	0.238
Status post sinus lift	0.998	^a^
No finding	0.803	1.22

Shown *p* values define the degree of agreement

^a^Describes an odds ratio close to zero or infinity due to numerical problems

* p < 0.05

### Inter-observer differences

Good inter-observer reliability [13] between the two consultants analyzing the CBCT scans was confirmed by Kappa coefficient (0.7) and McNemar’s test (*p* > 0.05).

**Fig. 2:**
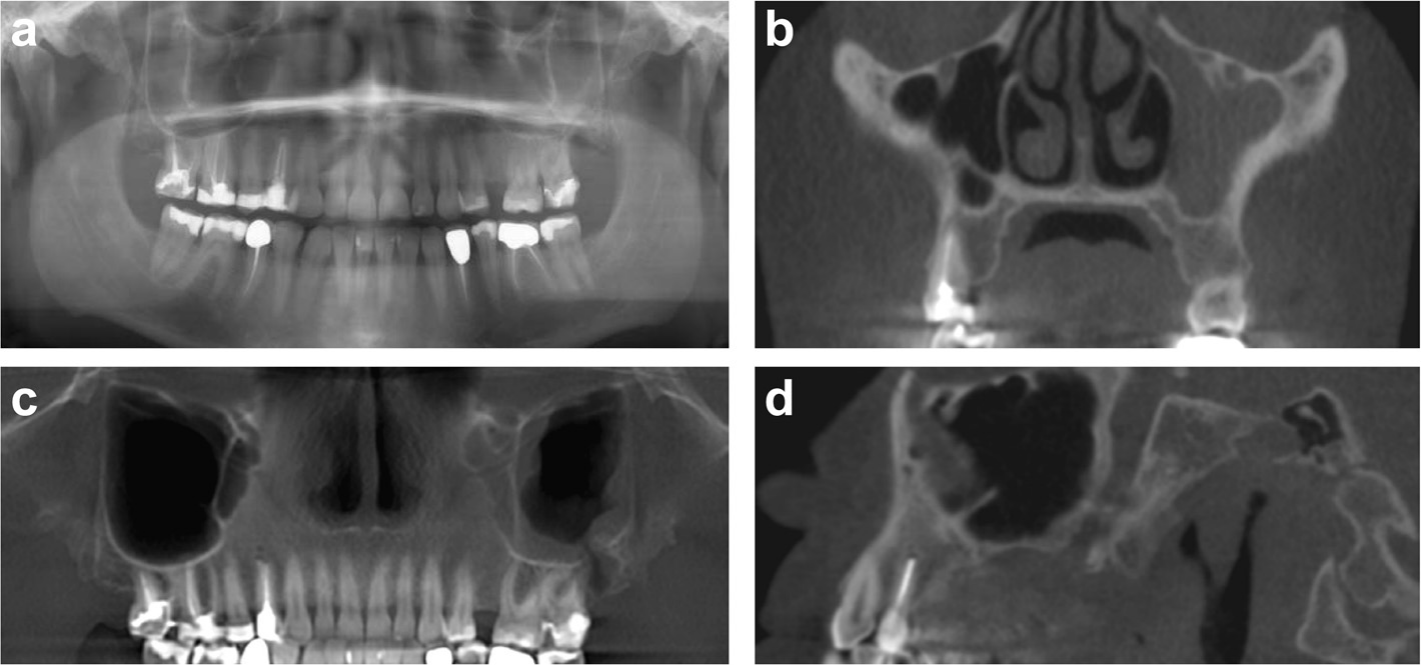
Radiographic findings of patient 19: oro-antral communication, basal opacity, basal septa; **a** panoramic radiography (Soredex, Cranex); **b–d** CBCT images (KaVo 3D eXam): coronal plane (**b**); panoramic reconstruction view (**c**); CBCT sagittal plane, right sinus (**d**)

The comparison of the two resident groups (first-year, last-year) examining 2D panoramic images showed that basal septa were significantly often rated differently by these two groups (*p* = 0.004, OR = 0.542). Further, it was shown that there is a significant good inter-observer agreement in detecting complete opacities (*p* < 0.001, OR = 6.133) (Table 3; Fig. 2).

**Table 3:** Agreement in evaluating the maxillary sinus on panoramic images

	Inter-examiner		Intra-examiner	
	*p*	OR	*p*	OR
Complete shadow	<0.001*	6.133	0.549	1.387
Basal shadow	0.441	1.186	0.621	0.806
Foreign body	0.596	1.207	0.991	0.993
Oro-antral communication	0.842	1.083	0.432	0.547
Basal septum	0.004*	0.542	0.375	0.704
Polypoid mucosal thickening	0.052	1.748	0.060	0.404
Maxillary bone cyst penetrating into the sinus	0.628	0.855	0.044*	0.331
Fluid level	0.653	1.515	0.999	0.000
Status post sinus lift	1.000	1.000	0.696	0.765
No finding	0.511	0.866	0.280	0.562

Inter-examiner describes agreement between two rating groups (first-year and last-year residents) and intra-examiner describes individual agreement between two evaluation runs. Shown *p* values define the degree of agreement

* p < 0.05

### Intra-observer reliability

The intra-observer comparison showed that assessors of panoramic radiographs were largely reliable considering the two evaluation runs of the same 2D panoramic images with a 4-week interval in between. The analysis indicated a significant lack of reliability (*p* = 0.044, OR = 0.331) in diagnosing maxillary bone cysts penetrating into the sinus (Table 3).

### Prevalence of findings

**Fig. 3:**
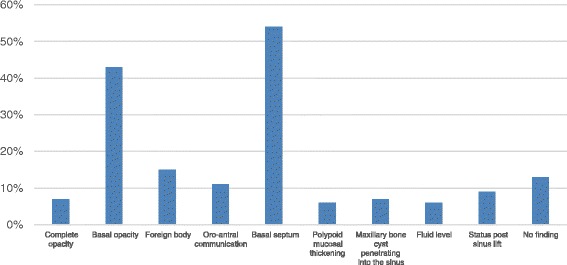
Descriptive illustration of 54 evaluated maxillary sinuses

On the basis of 54 evaluated maxillary sinuses, the most frequent radiographic findings in CBCT were basal septa (54 %), followed by basal opacities (43 %), and foreign bodies (15 %) (Fig. 3).

## Discussion

The purpose of this study was to analyze the validity of different anatomic variations and pathologies of the maxillary sinus found in 2D panoramic radiography by comparing them to those initially detected on CBCT images. Additional aims were the evaluation of inter- and intra-examiner differences on panoramic-radiograph-driven evaluation of the maxillary sinus.

### Differences between CBCT- and panoramic-radiograph-driven evaluations of the maxillary sinus

There is a moderate risk for false diagnosis of the maxillary sinus if only panoramic radiography rather than CBCT is used. In the present study, comparing 2D to 3D imaging, solely maxillary bone cysts penetrating into the sinus were frequently detected differently. Maestre-Ferrin et al. compared the efficacy of panoramic radiography, computed tomography (CT), and 3D CT in the diagnosis of mucosal thickening, mucous cysts, or complete opacity when using implant-planning software and showed that panoramic radiography was comparatively inferior [14]. Maestre-Ferrin et al. [14, 15] also showed that panoramic radiography led to false-positive and false-negative findings in the visualization of maxillary sinus septa in almost half of their cases, and Krenmair et al. [16] observed the same inaccuracy of panoramic radiography in detecting antral sinus septa in 13 out of 61 cases. Our study demonstrated no significant differences between 2D and 3D imaging methods in the detection of basal septa.

### Inter-observer reliability

The inter-observer disagreement between the two resident groups (first-year vs. last-year residents) examining 2D panoramic images was significant in the detection of the basal septa. As mentioned above, Maestre-Ferrin et al. have already indicated that panoramic radiography is insufficient for the detection of sinus septa [15], which complements to Shahbazian et al.’s finding that even though panoramic radiography provides a broad view of the sinus floor, it is unsuitable for detecting small lesions, due to low spatial resolution [11]. A similar observation was obtained by Dreiseidler et al. who confirmed superior visualization of all important high-contrast structures for CBCT compared to panoramic radiography with a focus on presurgical implant planning [17].

### Intra-observer reliability

There was only little intra-observer variation. The literature shows that the intra- and inter-examiner variation in the interpretation of radiographs may exceed the variation of imaging techniques and diagnostic yield [12, 18, 19]. That some variations may not be eliminated despite observer training has already been indicated by Kullman et al. [20]. Their study analyzed inter- and intra-observer differences in assessing panoramic radiographs with regard to radiographic bone height at two assessments several weeks apart. Both outcomes of two observers were described as reliable but not excellent though both raters were experienced.

One limitation of the latter and also of the present study may be the relatively small number of raters. Another limitation of this study may be the prevalence imbalance of different findings in the maxillary sinus resulting in a negative impact on our statistical calculation. This might include not only the low prevalence of maxillary bone cysts penetrating into the sinus but also the high prevalence of basal septa, an imbalance former studies have already demonstrated [15, 21, 22].

An explanation for our reported findings may be that, due to the superimposition of different structures, low spatial resolution and visual loss of cortical plates or undulating concavities, precise evaluation of a maxillary sinus finding is difficult in 2D panoramic radiography [5, 11]. Moreover, this difficulty might express that, as a consequence, the shown inter- and intra-observer variation in the interpretation of 2D radiographs may exceed the diagnostic yield [12].

Undiagnosed sinus conditions may be associated with chronic orofacial pain that is one of the most common reasons why patients consult physicians [23]. Moreover, precise assessment of the maxillary sinus by obtaining information on bone characteristics, on condition of Schneiderian membrane, on the presence of septa, and on the lateral sinus wall is mandatory prior to any lateral or internal sinus floor elevation [7, 8]. Currently, different radiographic means are used for preoperative tooth and bone-site and implant-site assessment. Clinicians commonly use 2D or 3D radiography. Both options imply advantages and disadvantages [4]. CBCT is used primarily to evaluate bony anatomy and to screen for overt pathology of the maxillary sinuses prior to dental implant treatment [24, 25]. However, prior to any radiographic imaging, especially for young patients, its benefit must be to weigh against its risk, with highest attention to the ALARA principle (*a*s *l*ow *a*s *r*easonably *a*chievable) [8]. This study indicates that panoramic radiography provides a sufficient view of the maxillary sinus for basic diagnostics, and it may be an adequate imaging method especially in the initial diagnostic phase. A precise assessment of different conditions of the maxillary sinus may only be possible using CBCT.

## Conclusions

The results of this study emphasize that panoramic radiography visualizes relevant findings of the maxillary sinus. In comparison to panoramic radiography, CBCT facilitates diagnosis of special conditions like penetrating cysts. The inter-observer comparison on panoramic radiographs demonstrated that basal septa were significantly often rated differently, and panoramic imaging may be based on a rather examiner-dependent assessment. Supplementary, the detection of maxillary bone cysts penetrating into the sinus with panoramic radiography showed a significant lack in reliability in the intra-observer comparison. Therefore, precise preoperative evaluation of the maxillary sinus on panoramic radiographs may be difficult. This could be relevant for consecutive surgical procedures; however, higher radiation dose and costs of three-dimensional imaging need to be considered.
